# Pathways of scientific input into intergovernmental negotiations: a new agreement on marine biodiversity

**DOI:** 10.1007/s10784-024-09642-0

**Published:** 2024-06-18

**Authors:** Ina Tessnow-von Wysocki, Alice B. M. Vadrot

**Affiliations:** Department of Political Science, University of Vienna, Kolingasse 14-16, 1090 Vienna, Austria

**Keywords:** International negotiations, United Nations, Marine biodiversity, BBNJ, Ocean protection, Science-policy interfaces

## Abstract

A new legally binding agreement for the conservation and sustainable use of marine biodiversity beyond national jurisdiction (BBNJ) was adopted by consensus on 19th June, 2023. Setting new regulations and filling regulatory gaps of the United Nations Convention on the Law of the Sea are expected to be informed by “best available science”. It is critical to understand how science entered the negotiations, which defined the global scientific knowledge base of decision-makers. This paper presents various pathways over which scientific input entered the BBNJ negotiations, using empirical data, collected through collaborative event ethnography, including participant observation and semi-structured interviews at the BBNJ negotiation site. Results show that scientific input in the BBNJ negotiations was not systematic and transparent but dependent on (a) available national scientific capacity, (b) financial resources, (c) established contacts and (d) active involvement of actors. Results of the study call for formalised science-policy interfaces, initiated by the UN Secretariat to guarantee a global knowledge base for decision-making. Keywords: international negotiations; United Nations; marine biodiversity; BBNJ; ocean protection; science-policy interfaces.

**Supplementary Information:**

The online version contains supplementary material available at 10.1007/s10784-024-09642-0.

## Introduction

A new legally binding agreement for the conservation and sustainable use of marine biodiversity beyond national jurisdiction (BBNJ) was concluded at the United Nations (UN) on March 4th and adopted on June 19th, 2023. The new instrument regulates access and benefit sharing of marine genetic resources, enables the establishment of area-based management tools, including marine protected areas, introduces obligations for the conduct of environmental impact assessments and strengthens capacity building and the transfer of marine technology in international waters (UNDOALOS, 2023). There seems to be general agreement that science needs to inform decision-making regarding marine biodiversity (Gjerde et al., 2019; Gownaris et al., 2019; Lubchenco & Grorud-Colvert, 2015; Marques & Carranza, 2013; Rabone et al., 2019). The UN Decade for Ocean Science exemplifies the need for and willingness of governments to engage with policy-relevant science throughout the development of ocean protection measures and new international agreements (Howell et al., 2021; Polejack, 2021; Ryabinin et al., 2019; Vadrot et al., 2022).

In past experience, however, the bargaining process of intergovernmental negotiations has been dominated by political and economic considerations, rather than by science (Ali & Susskind, 2014). There is vast literature on the role of science in international environmental agreements, including regime formation (e.g. Driesen, 2004; Haas, 1992), science-policy interfaces (De Donà, 2021; Koetz et al., 2012; van den Hove, 2007; Matsumoto et al., 2020; Schroeder et al., 2008), science advice of expert committees or formalised subsidiary bodies (Andresen, 2014; Andresen et al., 2018; Castells & Ravetz, 2001; Chasek, 2019; Kohler, 2019; Weiss, 2003), intergovernmental science bodies, such as the Intergovernmental Panel on Climate Change (IPCC) and the Intergovernmental Science-Policy Platform on Biodiversity and Ecosystem Services (IPBES) (Vadrot 2014 Hughes & Vadrot, 2019; Turnhout et al., 2016;Vadrot 2014), and scientific assessments (Selin & Eckley, 2003). Some scholars have used ethnographic approaches during formal negotiations but focused on science-related conflicts (e.g. De Santo et al., 2019; Gray et al., 2014; Hughes & Vadrot, 2019; Hughes et al., 2019). Regarding the BBNJ negotiations (Tessnow-von Wysocki & Vadrot, 2020), research looks at imbalances in academic authorship (Blasiak et al., 2016; Tolochko & Vadrot, 2021), science cooperation (Harden-Davies & Snelgrove, 2020), stakeholder perceptions on science-based approaches (Gaebel et al., 2020) and relevant traditional knowledge (Mulalap et al., 2020). Yet, there is a lack of research on ways over which science enters the BBNJ conferences and intersessional period. Our study therefore focuses on pathways of scientific input at the *negotiation stage*.

We understand science-policy interfaces as diverse forms of “exchanges, co-evolution, and joint construction of knowledge with the aim of enriching decision-making” and account for “relations between scientists and other actors in the policy process” (van den Hove, 2007). Thus, we emphasise *practices* by scientists and policy-makers within the BBNJ negotiations. We look at pathways of scientific input as *formal and informal practices that bring science into the negotiation process,* i.e. means by which science reaches the formal BBNJ negotiation setting and that is made available to state and non-state actors throughout the process of agreement-making. Formal practices refer to science-policy interactions, officially initiated or overseen by the UN Secretariat; informal practices are hereby defined as the science-policy interactions among—and on the initiative of—individual actors outside of the “formal” ways. In this light, we study *how*, *through which actors* and *under what conditions* science enters the BBNJ negotiations during the negotiation stage.

This article first introduces the state of the art of scientific input into intergovernmental negotiations and categorises the negotiation process into different stages (Chasek, 2001a, 2001b; Young, 1998), arguing that while limited scientific input into the *negotiation stage* has been criticised (Ali & Susskind, 2014), research has so far failed to open the blackbox of possible pathways and conditions thereof. Second, it lays out the methodological approach of collaborative event ethnography (CEE). Third, it identifies pathways of scientific input into the BBNJ intergovernmental negotiations and intersessional period and uncovers their conditional factors. Finally, it discusses findings and concludes by emphasising the need for a systematic inclusion of a diversity of actors, disciplines and knowledge systems when designing a new agreement for the governance of the global commons.

## The role of science in intergovernmental negotiations

Science has a significant role in agreement-making (Hughes & Vadrot, 2023; Hughes et al., 2021; Vadrot, 2020; Johnston, 2019). Particularly in the case of environmental issues, scientific input is crucial for decision-making, as these topics are often technical and complex (Haas, 1992; O'Neill, 2017; Weiss, 2003). Through science, environmental problems can be identified, understood and more effective responses be developed (Andresen, 2014; Castells & Ravetz, 2001; Meyer, 2016,2001). The importance of scientific input to inform policy decisions is highlighted by the UN system, acknowledging that “decision-makers need accurate scientific and technical information about the nature of threats, how each actor will be affected, and the types of arrangements that can be developed to address transboundary and global risks” (Chasek, 2019, p. 17).

The most influential concept to capture the role of science in International Relations (IR) continues to be the concept of “epistemic communities”, which emphasises the importance of organised networks of scientists and experts in shaping international environmental governance (Haas, 1992, 2014, 2016, 2017). What distinguishes epistemic communities from the broader idea of a scientific community are their specific characteristics: (a) “a shared set of normative and principled beliefs”; (b) “shared causal beliefs”; (c) “shared notions of validity”; (d) “common policy enterprise” (Haas, 1992, p. 3). It is assumed that scientific information from such networks supports decision-makers “to articulate an understanding of the world and of their own policies and interests” (Haas, 2016). Research on scientific input into international negotiations includes attributes necessary for linking science to policy, namely salience, credibility and legitimacy (Cash et al., 2003), conditions under which policy-makers regard science as relevant and use it in international negotiations (Dimitrov, 2006; Lidskog and Sundqvist, 2015; Rietig, 2014) and the role of consensual information (Kailis, 2017). While literature has initially drawn a clear line between science and policy (Ehrlich, 2006; Haas, 1992; Rose & Parsons, 2015; von Winterfeldt, 2013), the “scientist-policy maker dichotomy”, whereby scientists produce and certify the facts and policy makers ensure legitimacy, has increasingly been criticised (Jasanoff, 2004; Litfin, 1994; Saltelli & Giampietro, 2017). Scholars argue that such an approach is limited, and overlooks the “rich spectrum of actors and competences” (Saltelli & Giampietro, 2017), highlighting the need to also study struggle over environmental knowledge (Hughes & Vadrot, 2019; Vadrot, 2020) and “relations between scientists and other actors in the policy process”, considering “exchanges, co-evolution, and joint construction of knowledge” (van den Hove, 2007).

### Scientific input at different stages of intergovernmental negotiations

**Fig. 1:**
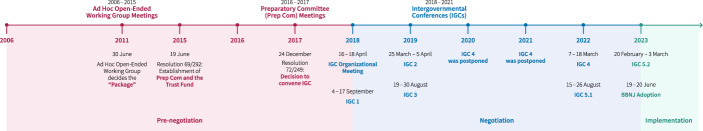
Stages of the BBNJ negotiations

Distinguishing between different stages of intergovernmental negotiations proves to be useful for analysing scientific input into agreement-making. In accordance with existing categorisations (Chasek, 2001a, 2001b; Young, 1998), we distinguish three stages of the BBNJ negotiations: the pre-negotiation (Ad Hoc Open-Ended Working Group & Preparatory Committee Meetings); negotiation (Intergovernmental Conferences IGCs); and implementation (after adoption of the agreement) (Fig. 1).

The first stage—the “pre-negotiation”—includes stakeholder input by scientific and technical experts, as well as informal discussions, in which Parties can analyse the situation, develop an information base and set the agenda (Chasek, 2001a, 2001b; Young, 1998). In the emergence of multilateral environmental agreements (MEAs), science is crucial to understand ecological systems and point to specific problems, such as the cumulative loss of biodiversity (O'Neill, 2017). On the basis of scientific input, international negotiations start informally, to decide whether the problem deserves global decision-making. In this regard, scientific publications and advocacy by experts can shape agenda-setting and influence how, when and which topics will be discussed (O'Neill, 2017).

The second stage is the formal “negotiation”, where negotiating of the actual terms of the treaty—the “bargaining”—begins (Ali & Susskind, 2014; Young, 1998). This stage serves to learn about other states’ positions, priorities and red lines, to make compromises and reach consensus. Scientists can shape the drafting of the final agreement by advising international organisations, national governments and non-governmental organisations (NGOs) and corporations during the negotiation stage (O'Neill, 2017, p. 106).

With consensus to adopt the new agreement, negotiators are in the final stage, namely the ratification and implementation (Chasek, 2001a, 2001b; Young, 1998). MEAs are implemented on regional and national levels, which requires the adaptation of national laws and can take several years. Science is crucial for the implementation of the agreement, as there is a need to monitor and review progress, evaluate the effectiveness and revise and adapt measures over time.

Our study focuses on the *negotiation stage, namely the intergovernmental negotiations and the intersessional period*. Limited scientific considerations at that stage have been criticised, as it decreases the likely effectiveness of the treaty in reversing ecological damage, arguing that “scientists have far too long been the missing link in the bargaining process” (Ali & Susskind, 2014). Our study opens the black box of scientific input into the bargaining process, identifies relevant actors and their formal and informal practices, and is applicable to intergovernmental negotiations beyond BBNJ.

## Data collection and analysis

This study uses data collected in the research project MARIPOLDATA 1 through collaborative event ethnography (CEE). An ethnographic research lens proves to be useful to empirically analyse the different ways through which science can enter the negotiations and potentially shape agreement-making. Considering negotiations as “sites” of agreement-making (Brosius & Campbell, 2010; Campbell et al., 2014a, 2014b; Gray et al., 2023; Hughes & Vadrot, 2019; Hughes & Vadrot, 2023; 2023; Marion Suiseeya & Zanotti, 2023; Vadrot, 2020; Witter et al., 2015), CEE enables us to study how, through which actors and under what conditions science entered the BBNJ negotiations during the *negotiation stage*.

The research team undertook in-person and digital CEE at the two-week-long conference sessions between 2018 and 2023.^2^ BBNJ negotiation sites included the plenary and informal working group sessions—where official bargaining took place—as well as other sites of interaction between scientists within the UN setting, such as side events and thematic workshops (Bansard, 2023; Schroeder & Lovell, 2012). Ethnographic fieldnotes used for this study include data collected in person, virtually or through a combination of the two for all IGCs, as well as the virtual intersessional work during the COVID-19 pandemic, coordinated by the UN Secretariat.^3^^,^^4^ The research team used a standardised format for fieldnote-taking, including a documentation of actors, their statements, and general observations—with indications to references to science (Vadrot et al., 2021a). Semi-structured interviews were conducted at the conference sites and digitally to trace practices of scientific input into BBNJ (Table 1).

**Table 1:** Qualitative Coding of Ethnographic Data

Data	Themes		Codes
Fieldnotes (Interventions in plenary and side events throughout IGC1-5.2)	Reference to Science	“Science “/ “Scientific “ Mentioning/explaining scientific concepts/terms Reference to scientific institutions Reference to scientific outputs/events Mention of scientific research activities & capacities	Reference to scientific publications Explanations of a scientific concepts/terms Statements on the importance of science for the agreement Invitations to scientific side events
Interview material (IGC2; 3; 5.1; 5.2)	Practices of scientific input	How the interviewee obtained or communicated scientific information Described broader practices of science-policy interfaces	Challenges involvement in parallel processes Meaning of science Role of science Scientific capacity Scientific concepts/terms Scientific cooperation Scientists in BBNJ negotiations

For analysis the data was limited to statements with a reference to science across all IGCs and online posts on the UN intersessional work platform. Interview material consisted of 70 interviews, including 29 state delegates; 7 representatives of intergovernmental organisations (IGOs); 19 representatives of NGOs; 12 natural scientists and 3 researchers from social sciences and law.^5^ The written transcripts of the interviews were inductively coded to portray the practices of scientific input, mentioned by interviewees and served to identify science-policy interfaces within the BBNJ process. At the final conference session, interviewees were confronted with the identified pathways to revise and complement the results.

## Results: pathways of scientific input into the BBNJ negotiation stage

**Fig. 2:**
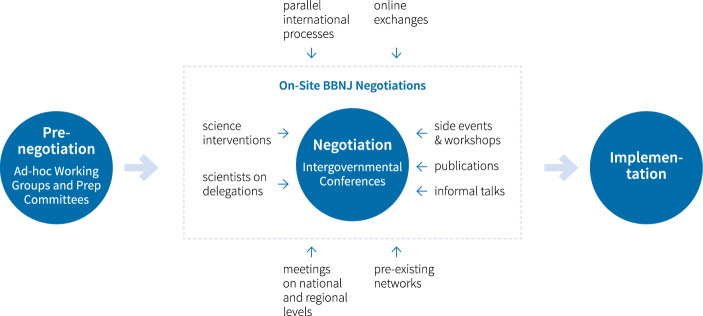
Scientific Input into the BBNJ Negotiation Stage

The *negotiation stage* in BBNJ consists of IGCs, happening in the UN Headquarters in New York, and the *intersessional period,* which is the time in-between the conferences. With a mandate of four IGCs to reach a legally binding agreement, the conferences started in 2018 and happened once to twice a year until in 2020 the last conference was postponed to 2022 and extended by two additional conference sessions.^6^ This section describes the pathways of *formal* and *informal* practices of scientific input into the BBNJ negotiations, as well as conditioning factors thereof, in both *on site* at the conferences and during the *intersessional period* (Fig. 2).

### Scientific input at the conference: on site

CEE gives insights into the structure and dynamics of the BBNJ negotiations on site. In the negotiations, nearly two-hundred state delegations negotiate the draft text, which is provided by the President of the Conference with support of the UN Division for Ocean Affairs and the Law of the Sea, based on previous discussions. Participants were registered as official state delegations, IGOs or NGOs. IGOs and NGOs were “observers” and granted different rights within the negotiations.^7^ Access to the main building also differed between these groups, permitting state delegates and NGOs with regular UN passes more time to stay on the premises than those holding a pass for the BBNJ meeting only. During the COVID-19 pandemic, the postponed fourth session restricted access to the negotiation rooms for observers.^8^ The final two sessions were in person, however, included small group discussions and president’s consultations that were closed to observers and not streamed online, oftentimes targeted at a certain group of state delegates only. The following section demonstrates formal and informal practices of scientific input on site.

#### Formal practices

##### Scientists on delegations

Scientists were formally represented on state or on non-state delegations. Scientist-based partnerships and initiatives informed the negotiations, such as the Global Ocean Biodiversity Initiative, and the Deep Ocean Stewardship Initiative (DOSI). These “scientific representatives”, however, blended in—whether individually or in an alliance—with other non-state actors, including environmental NGOs and business representatives. These three actor groups (science, NGO, private sector) were categorised under “NGOs” on the participant list and enjoyed the same speaking and access rights. Additionally, scientists could be on IGO delegations, such as on the Intergovernmental Oceanographic Commission of the United Nations Educational, Scientific and Cultural Organisation (IOC-UNESCO), advising the BBNJ process (Scientist_1_03/2019).

Scientists were also part of national delegations and represented particular states. In this case, they attended regular on-site coordination meetings in-between the formal sessions (State_Delegate_15_04/2019), which served as a ground for interaction between actors but were generally only accessible to state delegations. Apart from scientific advisors on delegations, some policy-makers were themselves scientists and entered the negotiations with knowledge of their previous or ongoing individual scientific research (e.g. Switzerland, IGC5.2).

Scientists are technically part of our delegation. So, when we feel that we need a heavy hand over scientists on board right here in New York we'll take one with us. (State_Delegate_28_08/2019)

Unless you're a very large delegation, […] specialists on every part and on the underlying issues, it is very difficult, if not near impossible, to engage substantively across the text. (State_Delegate_77_03/2023)

Interviewees emphasised differences among state delegations’ resources to have scientists on their delegations (IGO_44_08/2019; Researcher_60_03/2021).

Could we have more scientists on our delegations? Yes. If we had the resources. (State_Delegate_61_04/2021)

The inclusion of scientists on state and non-state delegations constitutes a formal practice of scientific input, as registration is enabled by the UN Secretariat. These examples demonstrate the variety of “hats” of scientists in the negotiations, sometimes even within the same conference. As shown, there are several ways for scientists to attend the BBNJ negotiations and bring science into the process. While non-presence of scientists on site does not exclude the possibility of the delegation having “their scientists” at home, advising them (State_Delegate_28_08/2019), interviewees pointed to the differences among states as regards scientific capacity and/or resources to include scientists on delegations.

##### Science interventions

Statements made in the negotiation room were simultaneously translated into all UN languages. Such interventions by state and non-state actors constitute another formal practice of bringing science into the negotiations. References to science in statements throughout all IGCs included: references to scientific publications, explanations of scientific concepts/terms, statements on the importance of science for the agreement, and invitations to scientific side events.

The World Ocean Assessments I & II (UN, 2016, 2021) served as scientific basis for statements, to portray awareness of the state of the ocean and draw conclusions for the role of the BBNJ instrument.^9^

We are acutely aware of the pressures arising from unsustainable usage of our oceans, which is supported by the findings of the first global integrated marine assessment, [quotes from the assessment][…] the impacts of climate change and other stressors arising therefrom which are resulting in ocean acidification, the warming of the ocean and the bleaching of our coral reefs. (Barbados on behalf of CARICOM, Plenary Statement, IGC1)

Delegates referred indirectly to science by mentioning own or other authors’ publications and information (UNEP, IGC1; ICO, IGC1),^10^ as well as advocating for side events on scientific topics (IOC-UNESCO, IGC1; Ocean Care, IGC1; Secretariat, IGC1).

UNEP stands ready to provide any technical input. (UNEP, Plenary Statement, IGC1)

Scientific input happened through scientists intervening themselves (Scientist_46_08/2019) to convey the relevance of their findings for the agreement. In other cases, scientists fed the science into interventions by communicating it through other actors (Scientist_14_04/2019). In small group and presidential consultations, “experts were brought into the room by delegations and […] give some clarity on a few issues.” (State_Delegate_77_03/2023).

BBNJ actors’ perceptions differed regarding the desirability of interventions by scientists during the negotiation stage, as seen in the following responses:

If you mean scientific input to the negotiation, there is not much because most of negotiators are lawyers or diplomats. […] otherwise it's going to be like a scientific talk. And that will require scientists as heads of delegations and they are the worst diplomats […]. (State_Delegate_28, 08/2019)

We need more [scientists] to come to the negotiations, rather than having diplomats, you know, removing the brackets, the commas, etc. We need somebody that can talk about substance. (State_Delegate_*44, 08/2019)*

The people who are at the microphone are not scientists and they probably shouldn't be either. (Advisor/State_Delegate _26_08/2019)

Intervening in the plenary sessions constitutes a significant formal practice of scientific input into the negotiations, as it is overseen by the UN Secretariat. CEE and interview data shows that state and non-state actors, including scientists and non-scientists, make scientific interventions throughout all IGCs. The extent to which science is brought into the negotiations, however, depends on the scientific capacities and willingness of actors on site, which is closely linked to their financial capacity to attend the conference.

##### Side events and workshops

Apart from representing a delegation and intervening in the plenary, scientists can also formally contribute to scientific input into the negotiations by presenting at a side event, seminar or workshop (Scientist_4_03/2019; Scientist_24_08/2019) that take place during lunchtime, in the evenings and on weekends, serving interaction between state delegates, scientists, and civil society actors. Side event hosts can invite scientists to give a talk (Scientist_23_08/2019; Scientist_43_08/2019; Scientist_42_08/2019; NGO/Scientist_46_08/2019).

We kind of used the side event to show governments or whoever is interested – decision-makers – that if you regulate, […] transboundary pollution within the agreement, that you cover a lot of threats that face the High Seas. (NGO_24_08/2019)

The lack of side events now […] removes an opportunity for scientists to be able to engage with […] their country representatives. (Scientist_76_03/2023)

Side events and workshops during the negotiation stage provided a space for scientists and other actors to communicate science that they perceived relevant for the negotiations. Non-state actors see side events as valuable spaces to “convey scientific concepts” (Advisor/State_Delegate_6_03/2019), “educate the delegates […] on specific topics or elements of the text, […] because some aren't fully up to speed with the BBNJ process” (NGO_21_08/2019) and “to make science relevant” (Scientist_24_08/2019).

Responses also reveal that these events serve to inform and are considered of “great importance to delegations” (State_Delegate_48_08/2019):

Side events are very useful […] I've picked up on some information that I've then fed to my delegation and then expressed from the floor. (State_Delegate_39_08/2019)

I look forward to [UNESCO side event] I'm sure there will be a lot of marine science there as well. That's what we need. (State_Delegate_27_08/2019)

Very informational […] very interesting and very thought-provoking. And of course, not being scientists ourselves, it's good to know what's actually happening in the field. (State_Delegate_61_04/2021)

Interviewees stated that they were approached after side events by interested BBNJ participants to receive the information presented (Scientist_23_08/2019) or were asked follow-up questions (Researcher_25_08/2019).

Despite these valuable existing initiatives, however, there are calls for a more systematic role of science in the negotiations:

Let's have something fully dedicated to science, back to back with the plenary – even before we discuss, […] a briefing so that when the delegates go to the plenary, they know what they're talking about.[…] the role of science must be better organised in this process. (State_Delegate_44_08/2019)

Results also indicate that while state delegates perceive side events as relevant, attendance is limited by time and capacity constraints, due to small delegations and parallel meetings (State_Delegate_28_08/2019; State_Delegate_38_08/2019; State_Delegate_39_08/2019). Some invited scientists were new, unknowledgeable or even misinformed about the procedures and their opportunity to get involved in the negotiations (Scientist_43_08/2019; Scientist_42_08/2019). This demonstrates the variety of scientists conducting relevant BBNJ research, who are not or only little aware about the negotiations and their opportunity to be involved. Broadly, this means that only scientists who were aware of the negotiations, motivated to engage in the political process and had the resources to attend, were presenting their findings at the negotiations at side events and workshops, to which not all delegations had access, due to resource and time constraints.

#### Informal practices

##### Scientific publications, briefs and other information material

A variety of documents were distributed during the conference on site, including publications, briefs, and information material that informally inserted science into the negotiations directly, by e.g. summarising main scientific concepts, or indirectly by advertising a scientific side event. Science can make its way into the negotiations through peer-reviewed articles, books and assessments, as well as publications in “grey literature”, such as reports by NGOs, or IGOs. Moreover, it can include policy briefs (Advisor/State_Delegate_6_03/2019), and technical briefs (Scientist_3_03/2019), generally written by NGOs, but also by policy-makers, advocating for a particular cause.

We have a paper […], but that might be too technical […]. So, we demystify these complex theories and we write them in a more accessible way. (Advisor/State_Delegate_6_03/2019)

Dissemination of publications, briefs and other information on site was mostly concentrated on a few locations in the UN building, where actors made their material available, in other cases, the material was put directly on the plenary desks of state and non-state actors. Ethnographers have noted the importance of such objects on site (“artefacts”) in meaning-making and interaction among actors. Information material ranges from small flyers^11^ to printed studies.^12^ Oftentimes, this material included colourful pictures of marine species and the ocean, though, occasionally, also shocking images, such as blood-covered sharks^13^ to illustrate the need for the new agreement to regulate the conservation and sustainable use of marine biodiversity.

Such artefacts allow scientists and policy-makers to interact directly. Peer-reviewed scientific articles, reports, technical and/or policy briefs were directly requested by state delegates or targeted to certain delegations or individual state representatives by scientists and NGOs. State delegates (NGO_21_08/2019; NGO_34_08/2019), as well as NGOs and IGOs contacted authors (NGO_34_08/2019).

In some cases, representatives of states were even directly involved in the *production* of publications. Some state delegates have been or are continuously undertaking scientific research and publishing in peer-reviewed scientific journals. These include individual researchers from the natural sciences and law as authors or co-authors of peer-reviewed literature, while being currently on delegations of CARICOM, the EU, Eritrea, Micronesia and Vanuatu (Broggiato et al., 2014, 2018; Hassanali, 2018, 2021; Hassanali & Mahon, 2022; Marciniak, 2017, 2020; Mulalap et al., 2020; Popova et al., 2019a, 2019b; Yadav & Gjerde, 2020). Connection between their published work and the state position they are representing varies and ranges from an explicitly stated separation between the research and the policy-making (EU) (Marciniak, 2020), to policy-makers publishing policy briefs and supporting the findings in their political statements (Eritrea; Palestine) (DOSI, 2023; Popova et al., 2019a, 2019b).

##### Informal talks

During the IGCs, informal meetings and spontaneous talks between state delegates and actors from academia and NGOs, including bilateral meetings and conversations over coffee, lunch or dinner, are an important pillar of the agreement-making process. On site at the negotiations, there are various spaces that invite for a chat, be it the “Vienna Café” directly outside of the plenary room, or other restaurants and cafes throughout the building, seating areas on several levels, inside and outside places to get together, as well as the “Delegates’ Lounge”, only accessible to state delegates and invited guests.

[…] like in every multilateral negotiation, there is a lot of informal personal contacts […] talks on the margins, lunchtime, coffee time […] We have one bilateral today with [an NGO]. (State_Delegate_19_08/2019)

Informal talks constitute a noteworthy practice of scientific input into intergovernmental negotiations. Such conversations are often informal gatherings but still constitute an integral part in bringing actors together, exchange ideas, explain political positions and offer an opportunity for the consideration of alternative arguments and scientific knowledge. It is also a way to extend networks for future collaborations but is again limited to the actors that attend the conference.

##### Reaching out to (pre-existing) contacts

Consultations with individuals from already established contacts constitute an additional pathway of informal scientific input into the negotiations.

We don't have that many native scientists […] I'm always happy to reach out to colleagues here in New York. […] sometimes they share papers with me […], the scientific aspects, and I try to incorporate them as much as possible. I don't formally consult them, but I have different colleagues, have a network, people can help me out sometimes. (State_Delegate_39_08/2019)

Interviewees from state delegations referred to consulting their own networks when in need for science. Existing contacts between scientists and policy-makers, thus, facilitate scientific input. Such networks play a role for the intergovernmental conferences, and more broadly for meetings throughout the intersessional period, in which state delegations are refining their positions. Moreover, recommendations for certain scientists and meeting scientists in the intersessional period have played a role in establishing new contacts to scientists (State_Delegate_77_03/2023).

### Scientific input during the intersessional period

The intersessional period offers an opportunity for state representatives to clarify views, re-calculate and refine their positions. This time was identified as particularly important for scientific input (State_Delegate_61_04/2021; Researcher_60_03/2021; NGO_41_08/2019), as state positions are formed prior to the conference and unlikely to change during the conference (NGO_41_08/2019; Researcher_60_03/2021) but that state delegations “maybe have some second thoughts, or [we] need to improve a specific position or idea for the next round” (State delegate_9_04/2019). State delegates emphasised that they “are taking it very seriously to work intersessionally [between the regional group members] and be able to come here with a ready-made position basically, and to be able to negotiate during those periods with other groups” (State_Delegate_38_08/2019).

#### Formal practices

##### Virtual intersessional “negotiations”

The intersessional period during the COVID-19 pandemic made online exchanges among and between state and non-state actors possible, which we consider “formal” when they were initiated by the UN Secretariat. As personal contact was reduced and in most areas of the world completely halted, the planned-to-be last negotiation session was postponed three times and extended by two further sessions. During this over two-year intersessional period, a formal online intersessional work platform was initiated by the UN Secretariat^14^ where registered participants to BBNJ could attend, post written responses to questions of the facilitators and comment on other actors’ suggestions and views (see Vadrot et al., 2022). State and non-state actors used this space to not only make references to scientific concepts and terms, but also to link or upload academic papers and evidence. In this way, the online space served to directly link statements to academic papers, policy briefs, assessments and video material and extended the traditional way of intervening with an oral statement.

#### Informal practices

##### Online exchanges

While during the intersessional period, actors regularly communicate via email, phone, social media and video calls (NGO_47_08/2019; State_Delegate/Scientist_19_08/2019), online interaction particularly increased since the extended intersessional period between IGC3 and IGC4 due to the COVID-19 pandemic (Vadrot et al., 2021a, 2021b). Intersessional meetings under Chatham House rules were organised by a few states and the High Seas Alliance (HSA),^15^ where actors could express their views but was not formally overseen by the UN Secretariat. These meetings were identified as important exchanges (Scientist_1_03/2019).

We're going to do monthly meetings to cover the range of issues […] to develop, further refine, and actually work through what we've heard in the High Seas Treaty Dialogues and during the informal intersessional interactions, […] whether or not we need to abandon certain things or refine or reach out to someone. (State_Delegate_61_04/2021)

While targeted invitations for some intersessional events resulted in limited participation and thus, information gaps between attendees and non-attendees (State_Delegate_77_03/2023), respondents generally supported online intersessional work, as online workshops and capacity building trainings were able to include a larger number of participants (with over 100 and 70 online participants in 2021, respectively), compared to a maximum of 80–90 and 25 participants in an in-person format (Researcher_60_03/2021_revised).

##### Preparing on national and regional levels

Prior to and after the IGC sessions, preparation on national and regional levels was taking place. State delegates referred to scientific publications in their preparation prior to the conference. Information sources also included reports of the Subsidiary Body on Scientific, Technical and Technological Advice of the Convention on Biological Diversity (CBD), the Nagoya Protocol, and the World Intellectual Property Organisation (WIPO) to understand the issue of MGRs (State Delegate_28_08/2019) and scientific reports by national research institutes (State_Delegate_9_04/2019).

Moreover, some state representatives actively sought inputs from the scientific community—which is however dependent on the engagement of the departments and agencies (State_Delegate_39_08/2019), as well as individual state representatives. Occasionally, state representatives contacted scientists directly for scientific explanations and briefings. Apart from existing contacts, individual scientists were recommended to state delegations, and new contacts established via webinars to reach out for information or even publish jointly (State_Delegate_77_03/2023).

Briefings and de-briefings between state delegates and scientists, such as ministry consultations, or meetings of already established national scientific committees, seminars, workshops and dialogues on national and regional levels are common (State_Delegate_40_08/2019), taking place in-between sessions of the IGCs, which can contribute to scientific input (Scientist_4_03/2019). Assuming previous contact with “their own scientists”:

Scientists may be not present. But the information has been given. And they influence of course, the positions of their delegations. (State_Delegate_33_08/2019)

State delegates mentioned they included Scientific Reports in their preparations and encouraged national experts to attend intersessional period meetings and comment on the national position from a scientific perspective (State_Delegate_9_04/2019). They also referred to “official scientific institutions” of their country or region that were considered in their preparations (9_04/2019; 28_08/2019) and in some cases scientists within the ministries (28_08/2019).

States invited experts to “regular periodic workshops” to “enlighten” them on “the relevant science and technology” (State_Delegate_9_04/2019). NGOs and governments jointly hosted workshops (NGO_21_08/2019; Researcher_60_03/2021) “to help the national delegates to prepare to get their positions together [regionally]” (NGO_47_08/2019).

These meetings on national and regional levels are instances for different stakeholder groups, including scientists, to discuss certain topics related to the new agreement. “Closed meetings […] between scientists, scientific institutions, ministries, the Navy, private sector in different aspects—shipping and others—fishing sector and lawyers” (State_Delegate_33_08/2019), organised by states, are taking place, as well as capacity building workshops, organised by NGOs and research institutes, bringing together state representatives from different countries and their respective responsible ministries, regional fisheries management organisations (RFMOs), global UN institutions, such as UNEP and civil society organisations with national regional and international experts and scientists (Researcher_60_03/2021). Trainings for negotiators that introduce lawyers and diplomats to the work in scientific labs are welcomed by state delegates, e.g. to discuss the treaty’s consequences (State_Delegate_33_08/2019) and described as “a real eye-opener for them” (Scientist_26_08/2019). But room for improvement remains, as a BBNJ scientist (76_03/2023) put it: “there needs to be more getting together of science and policy people, not just nationally, in terms of combining scientists and policy-makers from different ministries, but also internationally.”

##### Using science from other negotiation settings

Scientific concepts can be brought informally into the BBNJ negotiations from other fora (State_Delegate_40_08/2019). Other fora oftentimes include pre-negotiation meetings, but also previous or parallel political processes. In this way, science can enter the negotiations through the reference to existing law that refer to scientific concepts already enshrined in international law under previous conventions and protocols, or concepts that are being discussed in parallel political processes.

The parallel process in the CBD Nagoya framework, […] debates on in situ and ex situ […] in silico and digital sequence information. […] that debate sort of over-spilled into this process and also the discussions in parallel in WIPO on disclosure of origin and intellectual property rights in general also have their impacts into this process. (State_Delegate/Scientist_19_08/2019)

Further examples include the concepts of the ecosystem-based approach, the precautionary approach and compatibility that have been adopted under previous international agreements and were mentioned throughout the IGCs. This pathway can be used by state and non-state actors who are attending or following parallel processes and connect the contents of discussions. “Science spill-over”—which refers to science being used beyond one and traveling to another negotiation setting—thus happened with various terms in the BBNJ process, making it a significant pathway of scientific input. Such pathway could be formalised when being made formal (i.e. when the link is made explicit by the UN bodies) but is currently informal, as brought in through delegates in the negotiations.

## Discussion: the complex pathways of science into BBNJ

Scientific input into the BBNJ negotiations is critical to explain technical and complex topics related to marine biodiversity, including different ecosystems, species, and the state of the ocean. Scientists from different disciplines, including natural and social sciences, contribute to knowledge of policy-makers on deep-sea research and how these activities play out in practice. In turn, there is concern about the lack of scientific understanding of decision-makers, who are new to the topics, due to continuously changing posts.

Our results highlight formal and informal pathways of scientific input into the BBNJ IGCs and intersessional period. Collected data also shows that policy-makers obtained new scientific information throughout the negotiation stage of international agreement-making until the very end of the bargaining process, some of which have adapted their statements in the plenary accordingly. This proves the existence of scientific input throughout the negotiation stage and its relevance to shape the negotiations. Yet, despite the articulated need to base policy decisions on science, scientific input to guide the BBNJ intergovernmental conferences was only *voluntary and broadly formalised* (through the opportunity to register scientists on delegations and intervene in the plenary, as well as present in side events), and *conditioned* by national financial, technical and scientific capacities. Merely a fraction of the pathways was initiated by the UN Secretariat, and rather largely dependent on national structures (e.g. the decision whether or not to deploy scientists on delegations) or individual efforts (to inform themselves/others about scientific issues).

This is concerning, as our interview data underlines *inequalities between state delegations* regarding scientific expertise on the national level and financial resources to ensure scientific advice on their delegations in New York, which created an imbalance of scientific knowledge among state delegates from the start of official negotiations. Oftentimes, scientific input into the negotiations happened through recognised national scientific institutions or personal contacts between the scientists and policy-makers: whether *on site* at the negotiations, or through established networks of the policy-makers or the responsible ministries during the intersessional period. This practice points to the importance of perceived salience, credibility and legitimacy (Cash et al., 2003) of the scientists and scientific information by policy-makers to be considered for policy-making, but also calls for formalised ways to enable a comprehensive scientific base for all delegations.

There was *no official representation of science in the negotiation room*. Participating scientists from research institutes and universities were grouped under “NGOs”, indistinguishable from environmental activists and industry representatives on the sidelines of the negotiations, only allowed to speak after no further interventions by state delegates were requested. Further, they were themselves responsible for the registration and travel. A network of deep-sea scientists, with “shared normative commitments” (Haas, 1992, p. 19) constituted the “Deep Ocean Stewardship Initiative”, DOSI. This community worked on diverse ocean-related topics and was actively involved in the negotiations, published policy briefs and received attention by policy-makers. The fact that DOSI had their own delegation and took the floor as a coordinated group of experts qualifies them as an epistemic community in Haas’ (1992, p. 27, 35) sense of transmitting their ideas “in tandem with a set of causal and principled beliefs and reflect a particular political vision”. However, as our data shows, they are still a self-organised, self-funded network to send representatives, and without an officially UN-recognised “science” badge. Moreover, our research portrays pathways that go beyond this transnational epistemic community of DOSI, which are only visible when considering social interactions and individual actors in the negotiations (van den Hove, 2007). Scientific input into the BBNJ negotiations was largely initiated informally, by a number of people who were either scientists presenting and advocating for their findings to be included into the negotiations; or individual state or non-state actors who actively united policy-makers and scientists in workshops and side events. This indicates that scientific expertise by scientists who were neither contributing to global assessments, nor actively involved through coordinated scientific groups or individually and were not in contact with policy-makers, risked to be left outside of the discussions.

Results also emphasise *varying efforts of scientific input among state delegations.* Not only the number of scientists to advise national delegations differed, but also opinions on what was considered relevant knowledge. During the intersessional period, scientific input was identified as important for state delegations to clarify and re-think their positions. While interaction between policy-makers and scientists existed and national scientific institutions were mentioned as the primary scientific advice, it however varied among states and regions.

Moreover, results point to *different types of scientific input across the negotiation stage*, depending on the level of advancement in the negotiations. Towards the end of the negotiation stage, with the start of IGC4, we observed less formal scientific input on site, as the Secretariat did not allocate room for side events or observer interventions. While restrictions due to the COVID-19 pandemic might have played a role, assumingly, at that stage, delegates were expected to have a comprehensive scientific understanding of the issues, even though diplomatic staff turnover continued to happen until the last session. Negotiation time was prioritised over observer interventions, resulting in pathways of scientific input shifting almost exclusively to informal. While the beginning of the negotiation stage was marked by formal pathways of scientific input (through side events, workshops and interventions by non-state actors), towards the end, focus was rather set on political bargaining. Yet, as interview material shows, scientific input was required by delegations throughout the negotiations, which, towards the end happened solely informally, making informed negotiations more difficult for small delegations in parallel sessions and in case of diplomats turnover.

We can also differentiate between *in-person and online formats* of scientific input. New online tools and webinars to inform policy-makers can further contribute to science-policy interfaces within intergovernmental negotiations and offer new opportunities for scientific input in the intersessional period. Intersessional work, set up by the UN Secretariat and informal dialogues, organised by three states and the HSA,^16^ maintained momentum for the completion of the agreement, while the fourth conference was postponed due to the pandemic and policy-makers could not formally move the process forward without further guidance from the UN Secretariat. Online exchanges offered a useful space for scientists to submit and for policy-makers to access scientific information. However, the intersessional period did not officially advance the draft text and *in-person* discussions were required for formal negotiations (Vadrot et al., 2021a, 2021b) and while it technically enabled broader participation, it risked to create small hubs of scientific knowledge, only known among a fraction of attending delegates.

Lastly, stakeholders in BBNJ took on *different roles*: There are examples of professional diplomats who are scientists; there are scientists representing NGOs or IGOs, individual scientists that are attending the negotiations as presenters at side events to portray their work and coordinated scientists in alliances. Secondly, we observed that policy-makers and scientists appeared as authors of *scientific publications*, relevant to the BBNJ negotiations, which is a form of co-production of science and policy. Thirdly, scientific information for state delegates was not strictly only coming from peer-reviewed publications or global assessments, but also influenced by the *national/regional perception of credibility, legitimacy and salience* (Cash et al., 2003) and *personal contacts* between individual policy-makers and scientists. Scholarship of international relations, which has focused on institutionalised science-policy interfaces, thus, would benefit from looking at science-policy interfaces as social processes (van den Hove, 2007) and the agency of individual actors in the science-policy spheres when studying scientific input into intergovernmental negotiations in the negotiation stage.

## Conclusion and outlook

Undisputedly, scientific input is required for policy decisions on the future of marine biodiversity. Yet, *how*, *over whom* and *under what conditions* scientific input was happening in the BBNJ negotiations has not been studied by IR scholars. We categorised the BBNJ negotiations into three different stages (Chasek, 2001a, 2001b; Young, 1998), namely the pre-negotiation (Ad Hoc Open-Ended Working Groups and Preparatory Committee Meetings), the negotiation (Intergovernmental Conferences & Intersessional Period) and the implementation (after adoption). Based on CEE fieldnotes and interview data, we identified pathways for scientific input into the BBNJ *negotiation stag*e. These included pathways initiated and overseen by the UN Secretariat, namely (i) scientists on delegations; (ii) science interventions; (iii) side events and workshops; and informal ones: (iv) publications; and (v) informal talks. In the intersessional period, virtual “negotiations” constituted an UN-initiated, formal pathway and informal practices were (vi) science spill-over from other negotiations, (vii) pre-existing contacts; (viii) preparation on national and regional levels, and (ix) online exchanges. As shown, different actors (scientists, NGOs, IGOs, state representatives) used these pathways to communicate, disseminate or access scientific information throughout the negotiation stage and even contribute to generation of new knowledge.

Our results show that scientific input throughout the BBNJ negotiation stage existed but UN-initiated science-policy structures were limited. Scientific input into the BBNJ negotiations was only voluntary and broadly formalised, and conditioned by (a) national scientific capacity; (b) financial resources; (c) established contacts and (d) active involvement of individual actors. This left scientific input during the *negotiation stage* to the agency of individual actors, requiring willing and financially capable state delegations to include scientists on their delegations; and scientists to join the conference sessions on delegations or at side events; national and regional scientific and financial capacity and established contacts; and ultimately, self-organised individual advocates for science integration into policy-making, including scientific networks, such as DOSI. This points to the potential for the UN Secretariat to encourage further formal practices of scientific input throughout the negotiation stage in future intergovernmental negotiations for new legally binding agreements.

The majority of pathways included non-UN initiated, namely *informal* practices, i.e. interaction between actors outside of formal UN structures. Existing concepts within IR literature fall short of explaining these complex science-policy interfaces, including the changing and multiple roles of BBNJ actors. It is important to discuss these findings in light of the negotiations for the protection of a global common, which is to be governed by all and protected for future generations and nature in itself. Knowledge generation requires embracing the diversity of actors and knowledge forms to inform decision-making *during the negotiation stage*. When it comes to designing future regulations, it is crucial that all actors have access to a global knowledge base. Currently, scientific input is conditioned by scientific and financial resources, established contacts, and active involvement of actors.

We therefore suggest a more UN-initiated scientific input into the *negotiation stage.* This could be achieved through a formalised, independent and inclusive scientific expert group/panel to produce assessments on issues discussed, that is formally represented on site, available for consultancy, to answer questions and undertake research in areas of interest to the international community—also during the intersessional period. Different multilateral environmental negotiations approach science-policy interfaces differently and the idea of a scientific advisory board to negotiations is not entirely new, as seen with the United Nations Convention to Combat Desertification negotiations (Chasek, 2019); however, in that case, it was supposed to provide scientific guidance *before* the negotiation sessions to the Secretariat, and did not allow for interaction and response to arising questions *during* the discussions with delegates (Corell, 1999). Having a continuous guidance *during* the negotiations instead, which is incorporated into the core discussions—as opposed to being sidelined as additional meetings, sometimes happening simultaneously to bilaterals—would also avoid clashing commitments of delegates and raise the role of science in the negotiation stage. Hence, such group/panel could provide evidence for specific topics, attend the discussions to clarify practical implementation of the agreement and answer questions of delegates. Moreover, the group/panel would be present as a recognised scientific observer, formally detached from a certain country position.

By ensuring participation of Global South scientists and access to scientific information by all delegations, such a panel would broaden the scope of issues discussed and approach a more equal scientific playing field in the negotiation process. To guarantee that current imbalances are not replicated in such a body, initiatives would need to be coupled with increased funding for developing countries to attend negotiations and commitment to strengthen and maintain scientific and technical capacity in the long-term, through e.g. needs assessments and evaluation of capacity building and technology transfer, international collaborations in two-way partnerships and lasting regional and cross-regional networks of scientists in the Global South (Harden-Davies & Snelgrove, 2020; Harden-Davies et al., 2024). In this way, imbalances between state delegations as regards their scientific capacity and resources could be accommodated, a formalised representation of knowledge for the international community to inform decisions on the global commons further approached, and uptake of science and other knowledge forms facilitated.

In future intergovernmental negotiations, established entities could provide such scientific advice. Access to a scientific expert group that follows the political discussions and offers guidance throughout the negotiations stage would facilitate effective treaty design for meeting the objectives. Online and hybrid forms can be considered for continuous and inclusive participation of scientists across diverse disciplines, regions and knowledge systems. Additionally, it is important to include scientific assessments on the topic and incorporate new ideas for science-policy interfaces, such as the proposed Panel for Ocean Sustainability (Gaill et al., 2022).

To design a global agreement on areas that are beyond the jurisdiction of any state, a comprehensive and inclusive knowledge base is crucial. As shown, a continuous, global scientific support during the negotiation stage of BBNJ was lacking and attempted through formal and informal practices along the way. For implementation, however, scientific advice, tailored to the topics of the treaty is envisaged in form of a scientific and technical body (Gaebel et al., 2024). Several parts of the new treaty empower the new science body to assess, evaluate and give recommendations.^17^ However, details of the role of such a body, such as its functions and operation, including the selection process remain to be negotiated in the upcoming Conference of the Parties,^18^ which calls for further research on its structure, characteristics and powers in light of an informed, inclusive and just governance of the global commons.

## Supplementary Information

Below is the link to the electronic supplementary material.

Supplementary file1 (DOCX 723 KB)

## Abbreviations

ABNJ: Areas Beyond National Jurisdiction
BBNJ: Marine Biodiversity Beyond National Jurisdiction
CARICOM: Caribbean Community
CBD: Convention on Biological Diversity
CEE: Collaborative Event Ethnography
COVID-19: SARS-CoV-2 Corona Virus Disease 2019
DOSI: Deep Ocean Stewardship Initiative
ERC: European Research Council
HSA: High Seas Alliance
IGC: Intergovernmental Conference
IGO: Intergovernmental Organisation
IOC-UNESCO: Intergovernmental Oceanographic Commission of the United Nations Educational, Scientific and Cultural Organisation
IPCC: Intergovernmental Panel on Climate Change
IPBES: Intergovernmental Science-Policy Platform on Biodiversity and Ecosystem Services
IR: International Relations
MEA: Multilateral Environmental Agreement
MGR: Marine Genetic Resource
NGO: Non-governmental Organisation
UN: United Nations
UNDOALOS: United Nations Division for Ocean Affairs and the Law of the Sea
UNEP: United Nations Environment Programme
USA: United States of America
WOA I: First World Ocean Assessment
WOA II: Second World Ocean Assessment

## Footnotes

1 See:https://www.maripoldata.eu/.

2 IGC1 retrospectively through video recordings; IGC2 (March/April 2019), IGC3 (August 2021), IGC5 (August 2022) and IGC5.2 (February 2023) in person; IGC4 (March 2022) digitally due to COVID-19 restrictions and intersessional period between IGC3 and IGC4; and between IGC4 and IGC5 digitally.

3 Virtual intersessional work: https://www.un.org/bbnj/content/Intersessional-work.

4 Data collected from other online forums (not initiated by the UN Secretariat) was not included (e.g. BBNJ Informal Dialogues: https://bbnjinformals.org/).

5 For this publication we identify interview material in the format (actor, number of interview from general database, date of the interview). In some cases roles are not clearly defined, e.g. when there is an NGO advisor on a state delegation (Advisor & State Delegate).

6 IGC 5.1 and IGC 5.2.

7 Differences between state delegations and others include *interventions* (state delegations speak first) and *attendance* at “informal informals” sessions (only passive participation of a restricted number of five non-state actors).

8 All IGC4 sessions were virtually accessible for passive attendance of registered participants, however, criticism by NGOs and some state delegations resulted in opening access to the negotiation rooms in week 2.

9 Reference to WOA I: Belize, IGC1; Barbados on behalf of CARICOM, IGC1; Maldives, IGC1; High Seas Alliance, IGC1; IOC-UNESCO, IGC2; International Seabed Authority, IGC1; UNEP, IGC1; USA, IGC1. Reference to WOA II: Secretariat, IGC2; IGC4.

10 UNEP. (2017). Regional Seas Programmes covering Areas Beyond National Jurisdictions. https://www.google.com/url?sa=t&rct=j&q=&esrc=s&source=web&cd=&ved=2ahUKEwj-1dKBnev9AhWJc_EDHUF8D88QFnoECBAQAQ&url=https%3A%2F%2Fwww.un.org%2FDepts%2Flos%2Fbiodiversityworkinggroup%2FRegional_seas_programmes_ABNJ.pdf&usg=AOvVaw2TM-mgFSpsW8RrHc9MqXEU Cicin-Sain et al. (2018). Policy Brief on Capacity Development as a Key Aspect of a New International Agreement. on Marine Biodiversity Beyond National Jurisdiction (BBNJ). https://www.google.com/url?sa=t&rct=j&q=&esrc=s&source=web&cd=&ved=2ahUKEwiwjcbhnuv9AhVKQPEDHSCbA78QFnoECBQQAQ&url=https%3A%2F%2Fglobaloceanforumdotcom.files.wordpress.com%2F2018%2F08%2Fpolicy-brief-on-bbnj-capacity-development-dec-2018-email-version.pdf&usg=AOvVaw3AR_ZO-Ze6KbDbuvbVKy-N

11 “The deep is an essential part of a healthy functioning ocean that provides food and oxygen, regulates our planetary systems and sequesters carbon. […]”, deepsea conservation coalition, Flyer.

12 Ross Salazar, E., Jiménez Ramón, J.A., Castro Campos, M., Blanco Bolaños, M. 2019. The Thermal Dome of Costa Rica / Atlas. MarViva Foundation, San José. 108 pp. Online available: https://marviva.net/wp-content/uploads/2021/10/Atlas-Domo-Termico-Ingles-MarViva-web.pdf.

13 Greenpeace International, 2019, “Sharks under attack”, Report online available: https://www.greenpeace.org/international/publication/22700/sharks-under-attack/.

14 See information on the Virtual intersessional work of the Intergovernmental Conference on an international legally binding instrument under the United Nations Convention on the Law of the Sea on the conservation and sustainable use of marine biological diversity of areas beyond national jurisdiction (BBNJ intersessional work): https://www.un.org/bbnj/content/Intersessional-work

15 An Alliance of more than 50 NGOs; See information on the BBNJ Informal Dialogues: https://bbnjinformals.org/.

16 The BBNJ Informal Dialogues are organised by Monaco, Belgium, Costa Rica and the High Seas Alliance, an Alliance of more than 50 NGOs.

17 See: UNCLOS (2023) Art. 19 (5), (6), Art. 20, Art. 21 (5), (7), (8), Art. 22 (1), Art. 24 (3), (5), Art. 26 (3), (4), Art. 28 (3), Art. 29 (2), (3), Art. 31 (1) (ii), (iv), (v), (vi), (3), Art. 33 (3), (4), (6), (7), Art 36 (2), (3), Art. 37 (3), (4) (a), (c), (d), (e), Art. 38, Art. 49.

18 United Nations Division for Ocean Affairs and the Law of the Sea (UNDOALOS), 2023, Art.47 (4).
