# Supplementary material for: Pathways of scientific input into intergovernmental negotiations: a new agreement on marine biodiversity
Source: Int Environ Agreem. 2024 Jun 18;24(2-3):325–48. doi: 10.1007/s10784-024-09642-0 (PMC11424709; doi:10.1007/s10784-024-09642-0)
Supplement: Supplementary file 1 — Supplementary file1 (DOCX 723 KB) [file 10784_2024_9642_MOESM1_ESM.docx]

### *Supplementary Material*

###### *i) Supplementary Material: Publications at IGCs*

**Selection of academic articles and briefs distributed during IGCs**

| **Time of Distribution** | **Type of Publication** | **Name of publication** |
| --- | --- | --- |
| IGC 5.2 | Policy Brief | Climate and biodiversity beyond national jurisdiction. DOSI 2023 |
| IGC 5.2 | Policy Brief | Briefing Note on the common heritage of humankind and areas beyond national jurisdiction. Becker Lorca & Derrig 2023 |
| IGC5 | Academic Article | The diverse benefits of biodiversity conservation in global ocean areas beyond national jurisdiction Santos et al. 2022 |
| IGC3 | Academic Article | Fish slipping through the governance net, Crespo et al. 2019 |
|  | Policy Brief | 30x30 A Blueprint for ocean protection, Greenpeace, 2019. |
|  | Policy Brief | So far, yet so close: Ecological connectivity between ABNJ and territorial waters, Popova et al. 2019 |
| IGC2 | Policy Brief | POLICY BRIEF: Adjacency: How legal precedent, ecological connectivity, and Traditional Knowledge inform our understanding of proximity. Dunn et al. 2019 |
|  | Policy Brief | Mico. What is migratory connectivity? |
|  |  |  |

###### *ii) Supplementary Material: UN Directory*

*
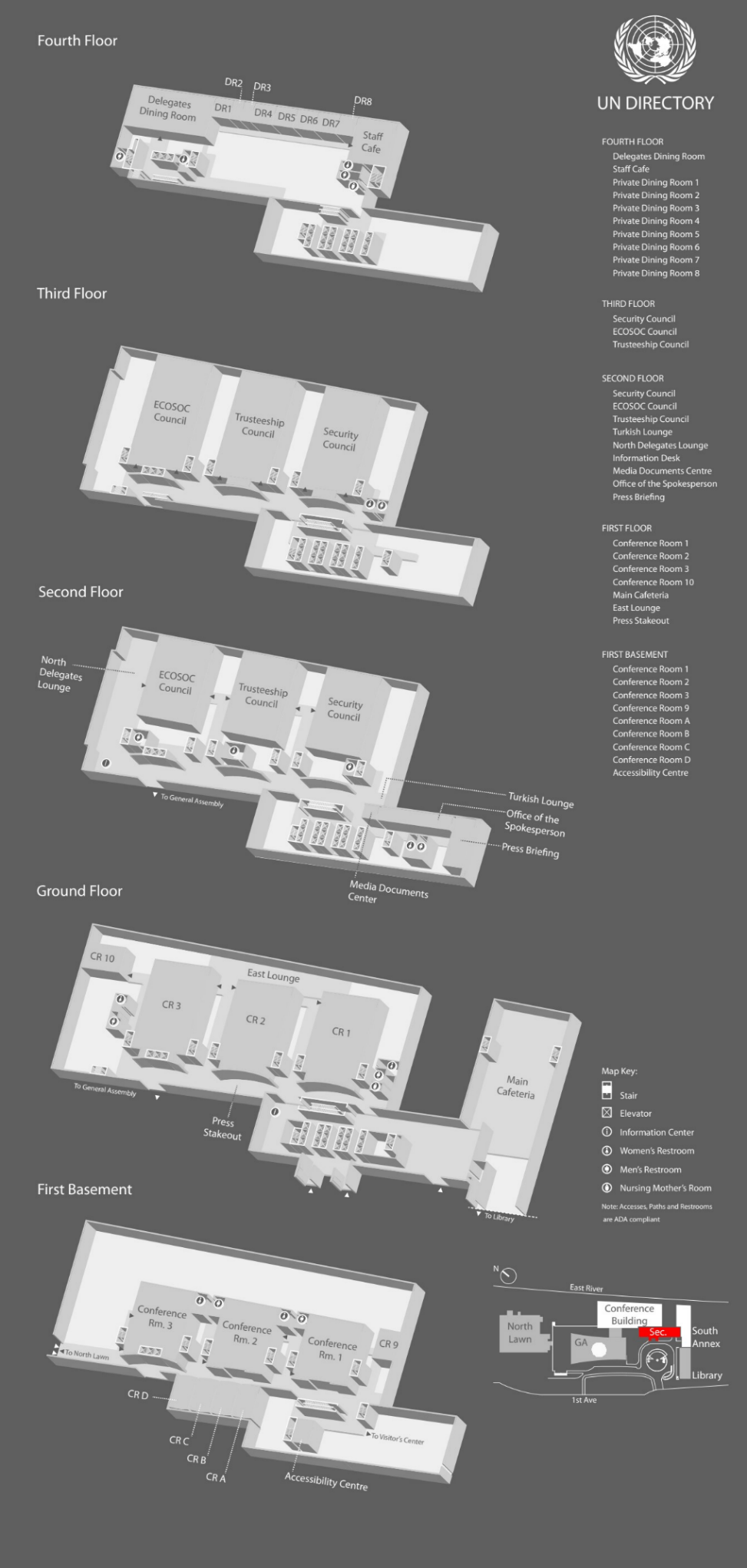
*

*Source: UN Statistics Division.* [*https://ggim.un.org/meetings/GGIM-committee/9th-Session/documents/GA_Building_Layout.pdf*](https://ggim.un.org/meetings/GGIM-committee/9th-Session/documents/GA_Building_Layout.pdf)
